# Synthesis and Photovoltaic Properties of 2D-Conjugated Polymers Based on Alkylthiothienyl-Substituted Benzodithiophene and Different Accepting Units

**DOI:** 10.3390/polym10030331

**Published:** 2018-03-18

**Authors:** Xunchang Wang, Chang Cheng, Yuda Li, Feng Wang

**Affiliations:** 1 Key Laboratory for Green Chemical Process of Ministry of Education, Wuhan Institute of Technology, Wuhan 430073, China; wang_xc@qibebt.ac.cn (X.W.); cc21029648@gmail.com (C.C.); psydli@163.com (Y.L.); 2 Hubei Novel Reactor & Green Chemical Technology Key Laboratory, Wuhan Institute of Technology, Wuhan 430073, China

**Keywords:** conjugated polymers, polymer solar cells, inverted structure, power conversion efficiency

## Abstract

Two new low bandgap conjugated polymers, PBDTS-ID and PBDTS-DTNT, containing isoindigo (ID) and naphtho[1,2-*c*:5,6-*c*′]bis[1,2,5]thiadiazole (NT), respectively, as an electron-deficient unit and alkylthiothienyl-substituted benzodithiophene (BDTS) as an electron-rich unit, were designed and synthesized by palladium-catalyzed Stille polycondensation. Both polymers showed good thermal stability up to 330 °C and broad absorption ranging from 300 to 842 nm. Electrochemical measurement revealed that PBDTS-ID and PBDTS-DTNT exhibited relatively low-lying highest occupied molecular orbital energy levels at −5.40 and −5.24 eV, respectively. These features might be beneficial for obtaining reasonable high open-circuit voltage and high short-circuit current. Polymer solar cells (PSCs) were fabricated with an inverted structure of indium-tin oxide/poly(ethylenimine ethoxylate)/polymer:PC_71_BM/MoO_3_/Ag. As a preliminary result, the PSCs based on PBDTS-ID and PBDTS-DTNT exhibited moderate power conversion efficiencies of 2.70% and 2.71%, respectively.

## 1. Introduction

Nowadays, polymer solar cells (PSCs) as a promising clean and renewable energy have attracted considerable attention owing to their manufacturing advantages such as light weight, low cost, flexibility, and ease of processing [1,2,3,4,5,6,7]. To enhance the photovoltaic performance of PSCs, numerous effective strategies have been performed to develop novel conjugated polymers containing electron-rich donor (D) and electron-poor acceptor (A) units with optimized highest occupied molecular orbital (HOMO)/lowest unoccupied molecular orbital (LUMO) energy levels. More encouragingly, PSCs with excellent power conversion efficiencies (PCEs) up to 13% have been reported [8].

Benzodithiophene (BDT) is one of the most widely used structural units for donor polymers in PSCs because of its rigid and large planar structure [8,9,10,11,12,13,14,15,16,17,18,19,20,21,22,23,24,25,26,27,28]. Recently, side chains of BDT-containing conjugated polymers have been found to play a critical role in affecting polymer packing and bulk heterojunction (BHJ) morphology. In comparison with alkoxy functionalized BDT, the conjugated polymer with alkylthienyl functionalized BDT can effectively tune the solubility, absorption bands, electronic energy levels, and carrier mobilities [29]. Alternatively, the sulfur atom possesses π–acceptor ability because of the unique p_π_(C)–d_π_(S) orbital overlap between the alkylthio substitution and the –C=C– double bond [30]. Therefore, the introduction of an alkylthiothienyl side chain to the BDT unit is for an important issue in BDT-containing conjugated polymers that could determine their photovoltaic performance. The two-dimensional (2D) BDT-thieno[3,4-*b*]thiophene (TT) copolymer reported by Li et al. achieved a PCE of 8.42% with a high open-circuit voltage (*V*_OC_) of 0.84 V. The enhanced *V*_OC_ value of 0.84 V for the PSC, achieved by introducing an alkylthiothienyl side chain, should arise from the down-shifted HOMO energy level of the polymer [30]. Subsequently, Hou and coworkers synthesized a novel alkylthiothienyl-substituted polymer PBDTTS1, and a higher PCE of 9.48% was achieved by replacing the branched alkyls (2-ethylhexyl) with linear alkyls (octyl), resulting in a superior absorption spectrum and enhanced hole mobility [17]. Recently, Sun et al. designed and synthesized a wide bandgap copolymer, PBT1-EH, which was optimized by an alkylthiothienyl side chain and thus a PCE of 10.6% was obtained [31]. These reports inspired us to rationally design a new alkylthiothienyl-substituted BDT-based conjugated polymer for high performance PSCs with high *V*_OC_ and short-circuit current (*J*_SC_).

As for the electron-poor acceptor units in the D−A photovoltaic polymers, isoindigo (ID) has attracted much attention because of its strong electron-withdrawing ability and the inherent advantages of its two indolone structures [21,32,33,34,35,36,37,38,39,40,41,42]. Naphtho[1,2-*c*:5,6-*c*′]bis[1,2,5]thiadiazole (NT), a benzothiadiazole-fused heteroaromatic unit, exhibited potential application in designing D–A conjugated polymers due to its thermal stability, broad optical absorption, and high electron-withdrawing effect [38,39,43,44,45,46,47,48,49,50,51,52,53].

**Scheme 1:**
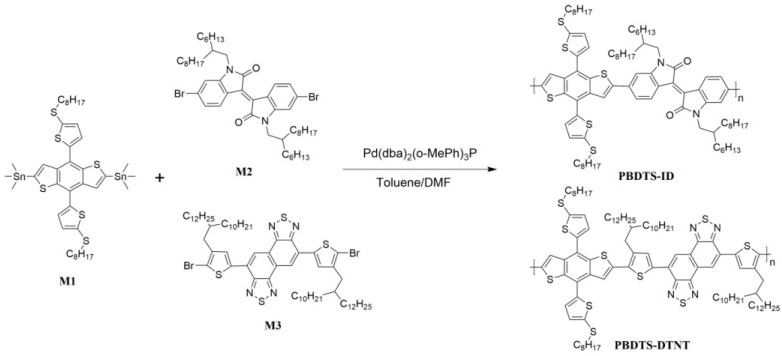
Synthetic routes for the polymers PBDTS-ID and PBDTS-DTNT.

Herein, we designed and prepared two novel D−A polymers, PBDTS-ID and PBDTS-DTNT, with alkylthiothienyl-BDT (BDTS) as a donor unit and ID or NT as an acceptor unit (see Scheme 1). The thermogravimetric analysis (TGA), UV-Vis absorption spectroscopy, electrochemical cyclic voltammetry (CV), and morphological properties of the polymers PBDTS-ID and PBDTS-DTNT were systematically characterized. The two polymers exhibited a deeper HOMO energy level of −5.40 eV for PBDTS-ID and −5.24 eV for PBDTS-DTNT, and broad absorption in the wavelength range from 300 to 842 nm. As a result, PSCs fabricated with PBDTS-ID as a donor and PC_71_BM as an acceptor exhibited a high *V*_OC_ of 0.84 V, leading to a moderate PCE of 2.70% under the excitation of standard AM 1.5 G spectrum at 100 mW·cm^−^^2^. Meanwhile, the *V*_OC_, *J*_SC_, and PCE of the PSCs based on the optimized fabrication conditions were 0.72 V, 8.31 mA·cm^−^^2^, and 2.71%, respectively, for the PBDTS-DTNT polymer.

## 2. Materials and Methods

### 2.1. Materials

All reagents were obtained from Aladdin Reagent Co. Ltd. (Shanghai, China), and Sigma-Aldrich Co. (Shanghai, China), and were used without further purification. Tetrahydrofuran (THF) and toluene were freshly distilled from sodium under a nitrogen atmosphere for use. Monomers M1, M2, and M3 were prepared and characterized according to the published procedures [17,34,50].

### 2.2. Characterization

Nuclear magnetic resonance (NMR) spectra were collected on a Bruker Advance III 600 (Bruker Co., Rheinstetten, Germany) with tetramethylsilane as a reference. Elemental analysis was carried out on a Vario EL III CHN elemental analyzer (Elementar Co., Langenselbold, Germany). Weight-average (*M*_w_) and number-average (*M*_n_) molecular weights of the polymers were determined on an Agilent in THF using a calibration curve of the polystyrene standards (Milford, MA, USA). The thermal properties of the polymers were evaluated on a TA instrument Q600-0825 (Waters Ltd, Hertfordshire, United Kingdom) under a nitrogen atmosphere with a heating rate of 10 °C·min^−^^1^. Powder X-ray diffraction patterns were obtained on a Bruker D8 Advance (Bruker Co., Karlsruhe, Germany). Absorption spectra were recorded on a UV-3100PC spectrophotometer (Shimadzu Co., Kyoto, Japan). CV was carried out on a CHI electrochemical workstation (Chenhua, Shanghai, China), and sn Ag/AgCl electrode, platinum wire, and glassy carbon were used as the reference electrode, counter electrode, and working electrode, respectively. The atomic force microscopy (AFM) patterns were obtained on a NanoScope NS3A system in tapping-mode (Veeco Inc., Santa Barbara, CA, USA). The transmission electron microscopy (TEM) patterns were obtained on a HITACHI H-7650 electron microscope using a 100-kV acceleration voltage (Hitachi Co., Tokyo, Japan).

### 2.3. Device Fabrication

The PSCs were fabricated with the structure indium-tin oxide (ITO)/poly(ethylenimine ethoxylate) (PEIE)/polymer:PC_71_BM/MoO_3_/Ag. The ITO glasses were cleaned with a surfactant scrub, and washed sequentially by deionized water, acetone, and isopropanol. Oxygen plasma treatment was conducted on the ITO substrates for 5 min. A PEIE thin layer was spin-coated on pretreated ITO-coated glass substrates and annealed at 100 °C in air for 5 min, after which the polymer:PC_71_BM (different weight ratio) solution was spin-coated onto the PEIE layer at a concentration of 6 mg·mL^−^^1^. After drying the solvent, MoO_3_ (10 nm) and Ag (100 nm) were successively evaporated through a shadow mask on top of the active layer, under a vacuum of 10^−^^5^ Pa. The current density–voltage (*J*–*V*) characteristics were recorded with a sourcemeter 2400 (Keithley Co, New York, NY, USA) controlled by a LabVIEW program in a nitrogen-filled glove box under white light illumination (100 mW·cm^−^^2^). The spectral responses of PSCs were recorded using a commercial external quantum efficiency (EQE)/incident photon-to-charge carrier efficiency (IPCE) setup (7-SCSpecIII, Beijing 7-star, Beijing, China).

### 2.4. Polymer Synthesis

#### 2.4.1. Synthesis of PBDTS-ID

M1 (193.7 mg, 0.2 mmol) and M2 (168.2 mg, 0.2 mmol) were dissolved in a mixed solvent of freshly distilled toluene (10 mL) and anhydrous *N*,*N*-dimethylformamide (DMF) (2 mL). The mixture was purged with argon for 10 min, and then Pd(dba)_2_ (3 mg) and tris(3-methoxyphenyl)phosphine (6 mg) were added into the flask as a catalyst. After being purged for another 10 min, the reaction mixture was heated to 110 °C for 24 h under an argon atmosphere. After cooling down to room temperature, the polymer PBDTS-ID was precipitated in the presence of methanol (150 mL). The precipitate was then subjected to Soxhlet extraction with methanol, hexane, and toluene. The polymer was recovered as a black solid (187 mg, 70.7%) from the toluene fraction by precipitation from methanol. Anal. calcd. for C_82_H_112_N_2_O_2_S_6_: C 72.99, H 8.30, O 2.37, N 2.07, S 14.25; found: C 72.80, H 8.44, O, 2.25, N 2.02, S 14.47.

#### 2.4.2. Synthesis of PBDTS-DTNT

M1 (193.7 mg, 0.2 mmol), M3 (247.2 mg, 0.2 mmol), Pd(dba)_2_ (3 mg), and tris(3-methoxyphenyl)phosphine (6 mg) were used to synthesize PBDTS-DTNT. The other experimental processes were the same as the preparation of the PBDTS-ID polymer described above. The target polymer, PBDTS-DTNT, was obtained as a dark solid (262 mg, 76.1%). Anal. calcd. for C_100_H_142_N_4_S_10_: C 69.79, H 8.32, N 3.26, S 18.63; found: C 70.32, H 8.24, N 2.95, S 19.03.

## 3. Results and Discussion

### 3.1. Synthesis and Characterization

**Figure 1:**
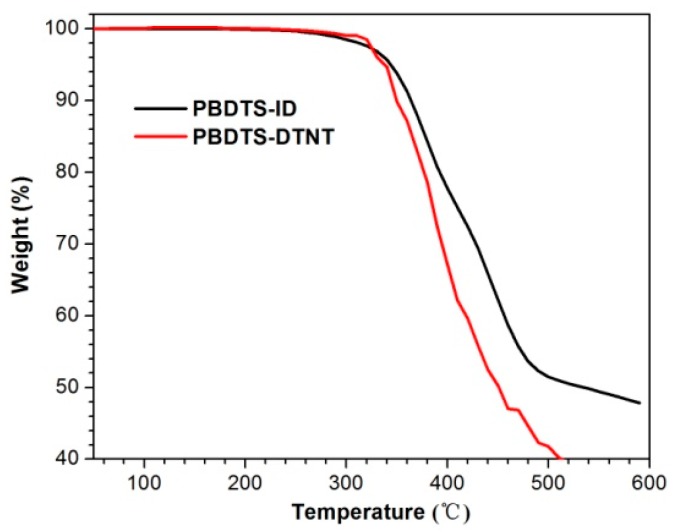
TGA curves of the polymers PBDTS-ID and PBDTS-DTNT.

M1, M2, and M3 were prepared according to the literature methods and their chemical structures were confirmed by NMR measurement (Figures S1−S3) [17,34,50]. The synthetic routes to the polymers PBDTS-ID and PBDTS-DTNT are outlined in Scheme 1. The polymers were prepared by Stille-coupling polymerization with distannyl and dibromide monomers using Pd(dba)_2_ and tris(3-methoxyphenyl)phosphine as a catalyst. Both polymers are soluble in normal organic solvent such as toluene, chlorobenzene, and *o*-dichlorobenzene. Gel permeation chromatography (GPC) was applied to measure the average molecular weights using THF at room temperature. The *M*_n_ of PBDTS-ID and PBDTS-DTNT was 23.4 and 47.2 KDa, respectively, with a polydispersity index of 1.78 and 2.10, respectively. Thermal properties of the two polymers were evaluated by TGA measurement (Figure 1), and the results suggested that both polymers exhibited good thermal stability, with decomposition temperatures (5% weight loss) of 348 and 335 °C for PBDTS-ID and PBDTS-DTNT, respectively.

### 3.2. Optical and Electrochemical Properties

**Figure 2:**
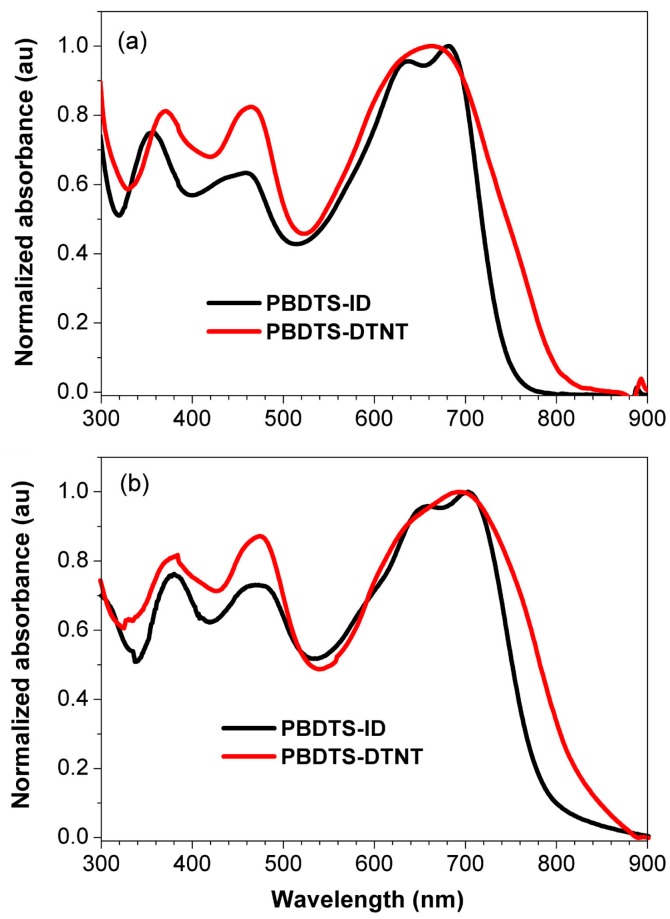
Normalized UV-visible absorption spectra of the polymers in chloroform solution (**a**) and thin solid films (**b**).

Figure 2 exhibits the normalized UV-visible absorption spectra of the polymers PBDTS-ID and PBDTS-DTNT in dilute chloroform solution and thin films, respectively. The corresponding absorption parameters are summarized in Table 1. PBDTS-ID showed a broad absorption profile in the range from 300 to 783 nm, with a maximum absorption peak (λ_max_) at 720 nm (Figure 2a). Compared to PBDTS-ID, PBDTS-DTNT displayed an obviously broadened and red-shifted spectrum extended to the longer wavelength region (~816 nm), which can probably be ascribed to the enhanced electron-withdrawing ability for the acceptors DTNT [38]. In the solid state, the polymers exhibited almost identical absorption profiles but broadened absorption regions, with λ_onset_ of absorption edge values at 803 and 842 nm for PBDTS-ID and PBDTS-DTNT, respectively, which is 20 and 26 nm red-shifted compared to that of their solutions [54]. The optical bandgaps (*E*_g_^opt^) of the PBDTS-ID and PBDTS-DTNT estimated from absorption edges in the solid state were 1.54 and 1.47 eV according to *E*_g_^opt^ = 1240/λ_onset_, respectively.

**Figure 3:**
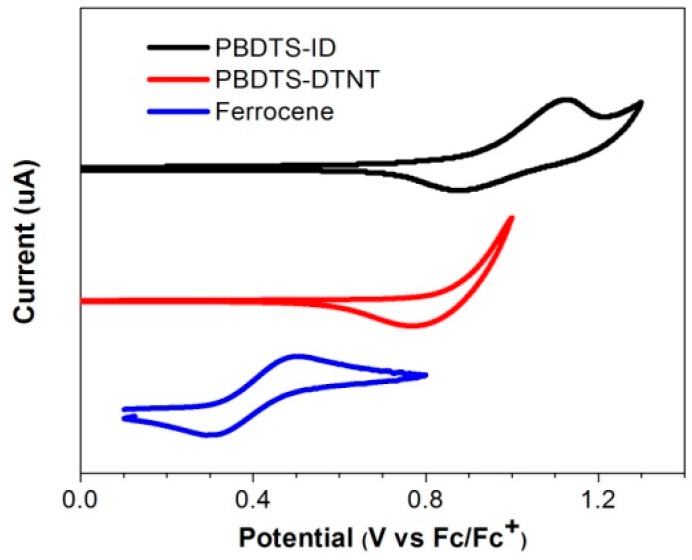
CV curves of the polymers PBDTS-ID and PBDTS-DTNT.

CV was employed to determine the energy level of the polymers in a three electrode electrochemical system. The acetonitrile solution containing 0.1 mol·L^−^^1^ tetrabutylammonium tetrafluoroborate (Bu_4_NBF_4_) was purged with argon for 5 min before CV measurement. The CV curve was obtained vs. the potential of an Ag/AgCl reference electrode, which was calibrated by the ferrocene-ferrocenium (Fc/Fc^+^) redox couple (−4.8 eV to vacuum). The redox potential of Fc/Fc^+^ in this study was found to be +0.37 V vs. the reference electrode. As shown in Figure 3, the onset oxidation potentials of PBDTS-ID and PBDTS-DTNT were observed at around 0.97 and 0.81 V, respectively. According to the empirical formula *E*_HOMO_ = −*e*(*E*_ox_^onset^ − *E*_1/2_^ferrocene^ + 4.8) (eV), the corresponding HOMO energy levels of PBDTS-ID and PBDTS-DTNT were calculated to be −5.40 and −5.24 eV, respectively (Table 1). The HOMO level of PBDTS-ID is deeper than that of PBDTS-DTNT, which should be beneficial for obtaining a higher *V*_OC_ in PBDTS-ID-based PSCs. This will be discussed in detail below. The LUMO energy levels of the polymers PBDTS-ID and PBDTS-DTNT were −3.86 and −3.77 eV, respectively, calculated from the empirical formula *E*_LUMO_ = *E*_HOMO_ + *E*_g_^opt^.

### 3.3. Hole Mobility

**Figure 4:**
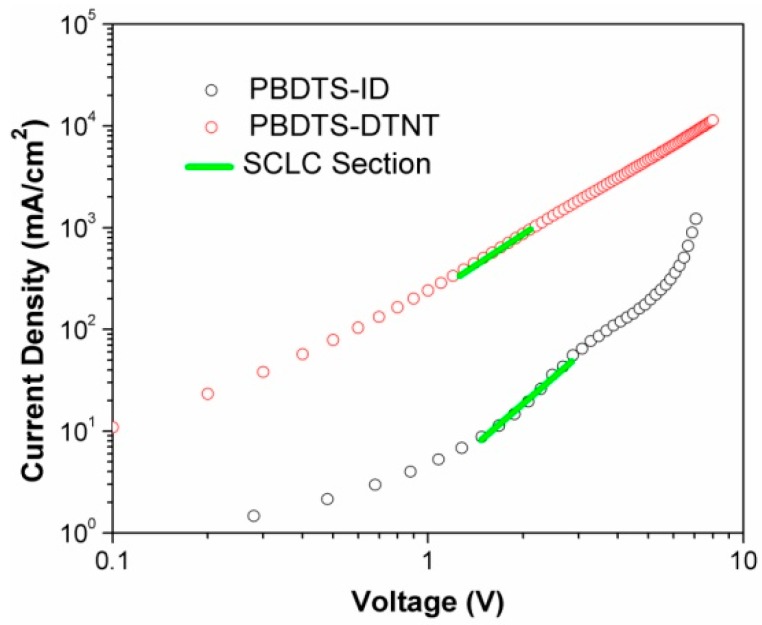
*J*–*V* curves of the hole-only devices under optimized conditions achieved by the space-charge-limited current (SCLC) method.

The hole-transport properties were a very important factor for polymer donor photovoltaic materials. To explore the hole mobility of the resulting polymers, we fabricated hole-only devices with a configuration of ITO/PEDOT:PSS/polymer:PC_71_BM/MoO_3_/Ag, and the hole mobilities were determined by using the space-charge-limited current (SCLC) method [14]. The hole mobility data were extracted by modeling the dark current and calculated from *J*–*V* characteristics fitted with the Mott–Gurney equation (Figure 4). The measured hole mobilities were determined to be 3.83 × 10^−5^ and 8.36 × 10^−5^ cm^2^·V^−1^·S^−1^ for PBDTS-ID:PC_71_BM and PBDTS-DTNT:PC_71_BM blended films, respectively (Table 2).

### 3.4. Photovoltaic Performance

**Figure 5:**
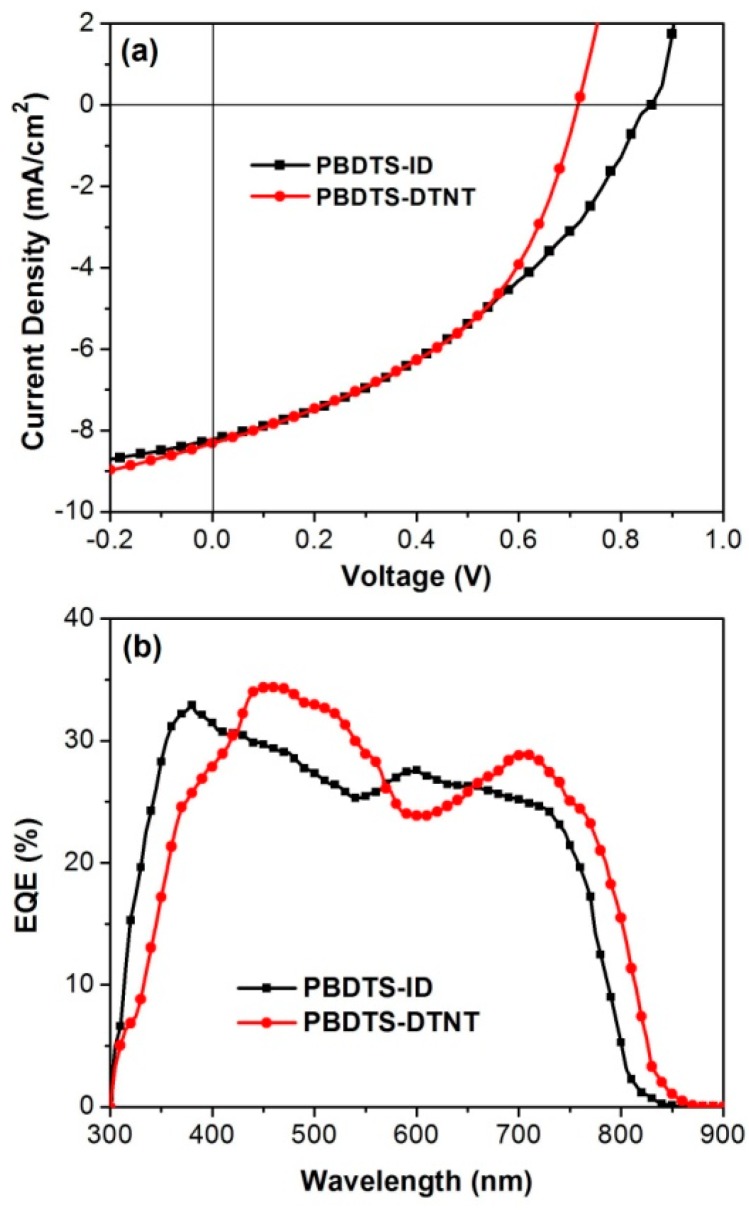
(**a**) *J*–*V* curves of the best-performing PSCs from the blend of the polymers and PC_71_BM; (**b**) external quantum efficiency (EQE) curves of the corresponding PSCs with 3% 1,8-diiodooctane (DIO) additive treatment.

A solution-processed PSC was fabricated with the invert architecture of ITO/PEIE (10 nm)/active layer/MoO_3_ (10 nm)/Ag (100 nm) to investigate the photovoltaic performance because the inverted device has demonstrated better ambient stability compare to the conventional device. The use of PEIE on the ITO substrate was found to improve the electron collection efficiency in the inverted device [55]. The active layer of the PSC was prepared from chlorobenzene blend solution of polymer and PC_71_BM. The optimized ratios of PBDTS-ID:PC_71_BM and PBDTS-DTNT:PC_71_BM were 1:1.5 and 1:2, respectively, with 3% 1,8-diiodooctane (DIO) as the additive. The current *J*–*V* characteristics are shown in Figure 5a and the key device parameters are listed in Table 2. The PSC based on PBDTS-ID exhibited a moderate PCE of 2.70% with *V*_OC_ = 0.84 V, *J*_SC_ = 8.21 mA·cm^−2^, and FF = 39.2%. Meanwhile, the PBDTS-DTNT-based PSC showed a similar PCE to the device of PBDTS-ID with a *V*_OC_ of 0.72 V, *J*_SC_ of 8.31 mA·cm^−2^, and FF of 45.8%. From photovoltaic performance, the PBDTS-ID exhibited higher *V*_OC_ than that of the device based on PBDTS-DTNT. In addition to the factors such as shunt resistance and recombination dynamics, the difference between the HOMO of the polymer and the LUMO of the PC_71_BM may also affect the *V*_OC_. Thus, the deeper HOMO energy level can result in the higher *V*_OC_ in the PBDTS-ID-based device. However, the PSC based on PBDTS-ID displayed a lower FF of 39.2%, which could be ascribed to the lower hole mobility of PBDTS-ID. Meanwhile, the broadened absorption of PBDTS-DTNT shown in the external quantum efficiency (EQE) spectra (Figure 5b), which covered a larger fraction of the sunlight, might be considered as the important factor for the enhancement of the FF for PBDTS-DTNT.

To verify the PCE of these devices, the EQE spectra of the devices under optimum conditions were investigated, as shown in Figure 5b. Both devices showed moderate photo-conversion efficiency in the wavelength range from 300 to 840 nm, with a maximum EQE value of 33% at 380 nm for PBDTS-ID and a maximum EQE value of 35% at 467 nm for PBDTS-DTNT, respectively. The current densities integrated from EQE values of PBDTS-ID and PBDTS-DTNT were consistent with the result obtained from the *J*–*V* measurements.

### 3.5. Morphology Study

**Figure 6:**
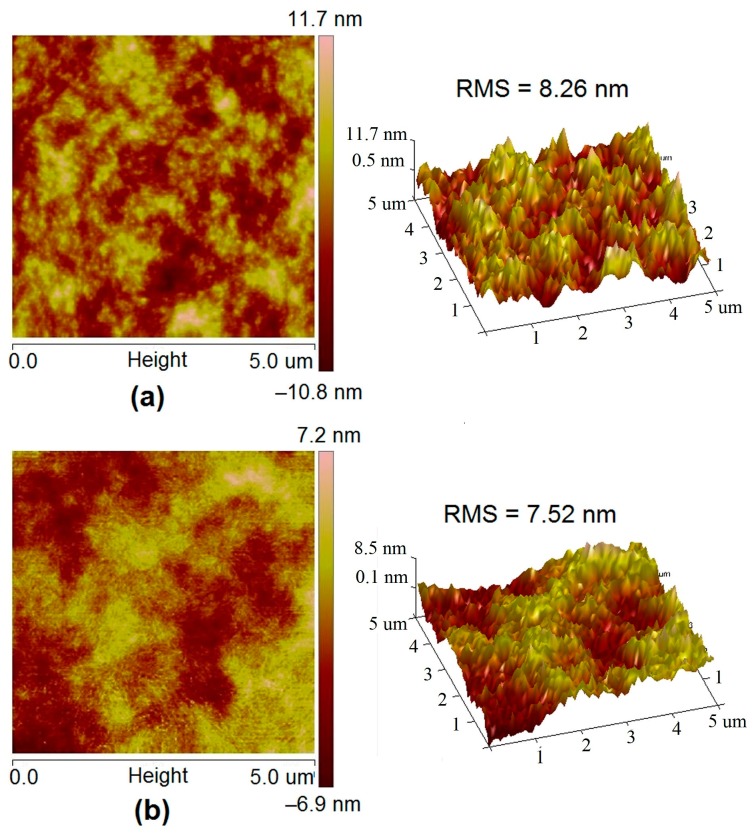
Atomic force microscopy (AFM) height images of PBDTS-ID:PC_71_BM (**a**) and PBDTS-DTNT:PC_71_BM (**b**) blend films with 3% DIO additive treatment.

In order to better understand the factors affecting the device performance, we investigated the surface morphologies of blend films using AFM and TEM measurements, respectively. The experimental procedure for the preparation of the active layer is identical to those used to fabricate the polymer and PC_71_BM blend film of the device. As shown in Figure 6, the active layers featured a grainy surface with high root mean square (RMS) roughness values of 8.26 and 7.52 nm for PBDTS-ID- and PBDTS-DTNT-based devices, respectively, suggesting serious phase separation. Under such conditions, photo-produced excitons cannot realize efficient dissociation and charge collection in the PSCs, causing an accompanied loss of photocurrent [14,18]. Therefore, the devices exhibited low *J*_SC_ and FF, and thus moderate PCEs.

**Figure 7:**
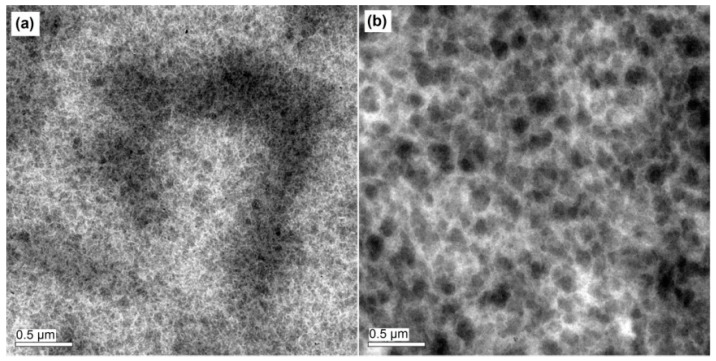
TEM images of PBDTS-ID:PC_71_BM (**a**) and PBDTS-DTNT:PC_71_BM (**b**) blend films with 3% DIO additive treatment.

The TEM measurement was applied to provide more information about the active layer. As shown in Figure 7a, larger fibrillar structures can be clearly found in the TEM image of the PBDTS-ID:PC_71_BM blend film, which might diminish exciton dissociation and charge transport efficiency and is not favorable device performance [10,56]. However, relatively smaller size aggregations were observed in the TEM image (Figure 7b) of the PBDTS-DTNT:PC_71_BM active layer compared to that of the PBDTS-ID:PC_71_BM blend film (Figure 7a), which is consistent with the observations obtained from the AFM data.

## 4. Conclusions

In this paper, we synthesized two polymers containing alkylthiothienyl-substituted BDT donating units and ID or NT accepting units through Stille-coupling polymerization. PBDTS-ID and PBDTS-DTNT exhibited a broad absorption range in the solar spectrum, and the calculated optical band gaps were 1.54 and 1.47 eV, respectively. The polymers are both thermally stable and were used as donor materials in combination with PC_71_BM in BHJ PSCs. The PSC devices prepared from PBDTS-ID and PBDTS-DTNT with the inverted structure of ITO/PEIE/polymer:PC_71_BM/MoO_3_/Ag showed maximum PCEs of 2.70% and 2.71%, respectively.

Overall, the photovoltaic performance of PBDTS-ID and PBDTS-DTNT was lower than that of many reported alkylthiothienyl-substituted BDT-based conjugated polymers, mainly because of the decreased current density. The positive results, such as suitable energy levels obtained for PBDTS-ID and PBDTS-DTNT, demonstrated that their photovoltaic performance might be increased by fine-tuning the structures of the conjugated polymers further.

## Figures and Tables

**Table 1:** Optical and electrochemical properties of the polymers.

Polymers	λ_onset_ (nm)	*E*_g_^opt^ (eV) ^1^	*E*_HOMO_ (eV) ^2^	*E*_LUMO_ (eV) ^3^
PBDTS-ID	803	1.54	−5.40	−3.86
PBDTS-DTNT	842	1.47	−5.24	−3.77

^1^ Bandgap estimated from the absorption edge in thin film (*E*_g_^opt^ = 1240/λ_onset_ eV); ^2^ The highest occupied molecular orbital (HOMO) level was estimated from cyclic voltammetry (CV) analysis; ^3^ The lowest unoccupied molecular orbital (LUMO) level was estimated by using the following equation: *E*_LUMO_ = *E*_HOMO_ + *E*_g_^opt^.

**Table 2:** Photovoltaic performance of the polymer solar cells (PSCs) fabricated by using the configuration of indium-tin oxide (ITO)/poly(ethylenimine ethoxylate) (PEIE)/polymer:PC_71_BM/MoO_3_/Ag.

Polymers	μ (×10^−5^, cm^2^·V^−1^·S^−1^)	*J*_SC_ (mA·cm^−2^)	*V*_OC_ (V)	FF (%)	PCE (%)
PBDTS-ID	3.83	8.21	0.84	39.2	2.70
PBDTS-DTNT	8.36	8.31	0.72	45.8	2.71

## Supplementary Materials

The following are available online at http://www.mdpi.com/2073-4360/10/3/331/s1, Figure S1: ^1^H NMR spectrum of M1 in CDCl_3_, Figure S2: ^1^H NMR spectrum of M2 in CDCl_3_, Figure S3: ^1^H NMR spectrum of M3 in CDCl_3_, Figure S4: X-ray diffraction patterns of blend films for the polymers.
