# Supplementary material for: Synthesis and Photovoltaic Properties of 2D-Conjugated Polymers Based on Alkylthiothienyl-Substituted Benzodithiophene and Different Accepting Units
Source: Polymers (Basel). 2018 Mar 18;10(3):331. doi: 10.3390/polym10030331 (PMC6414851; doi:10.3390/polym10030331)
Supplement: Supplementary file 1 [file polymers-10-00331-s001.docx]

***Supporting Information for***

Synthesis and photovoltaic properties of 2D-conjugated polymers based on alkylthiothienyl-substituted benzodithiophene and different accepting units

Xunchang Wang ^1^, Chang Cheng ^1^, Yuda Li ^1^ and Feng Wang ^1,2,^*

^1^ Key Laboratory for Green Chemical Process of Ministry of Education, Wuhan Institute of Technology, Wuhan 430073, China; wang_xc@qibebt.ac.cn (X.W.); cc21029648@gmail.com (C.C.); psydli@163.com (Y.L.)

^2^ Hubei Novel Reactor ＆ Green Chemical Technology Key Laboratory, Wuhan Institute of Technology, Wuhan 430073, China

***** Correspondence: psfwang@wit.edu.cn; Tel.: +86-27-8719-4980

**Table of Contents**

1. ^1^HNMR of the monomers M1−M3

2. X-ray diffraction patterns of blend films for the polymers.

1. ^1^HNMR oftheMonomers M1-M4


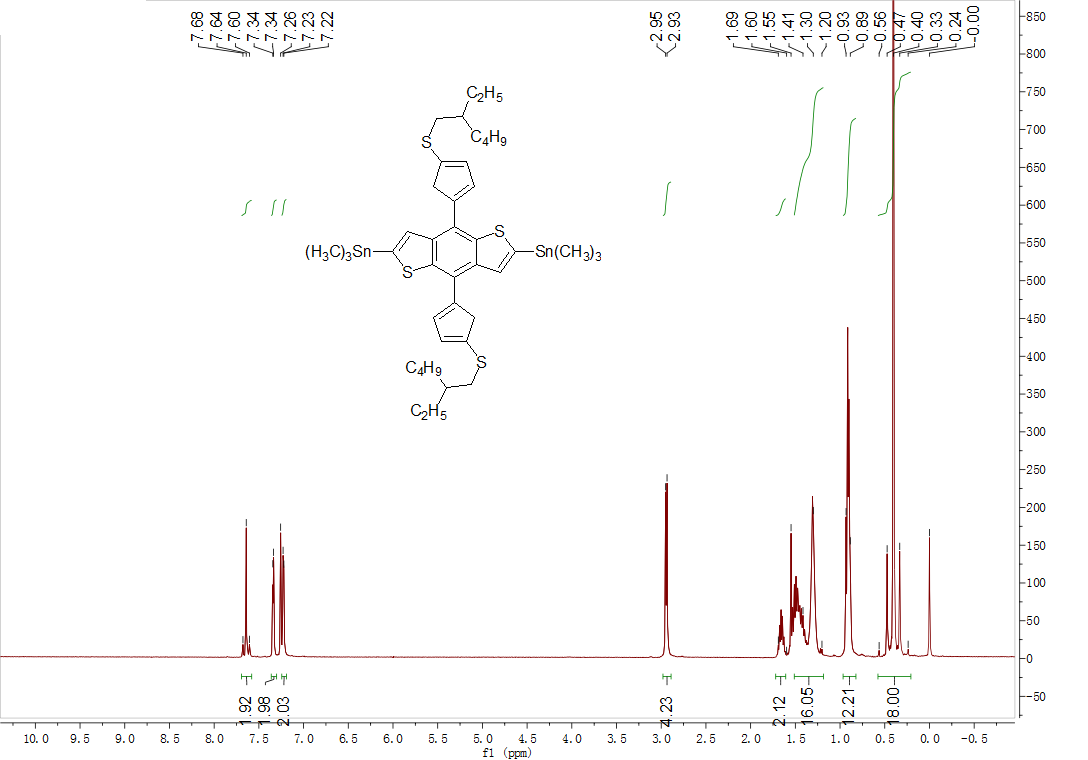


**Figure S1.**^1^H-NMR spectrum of M1 in CDCl_3_ solution.


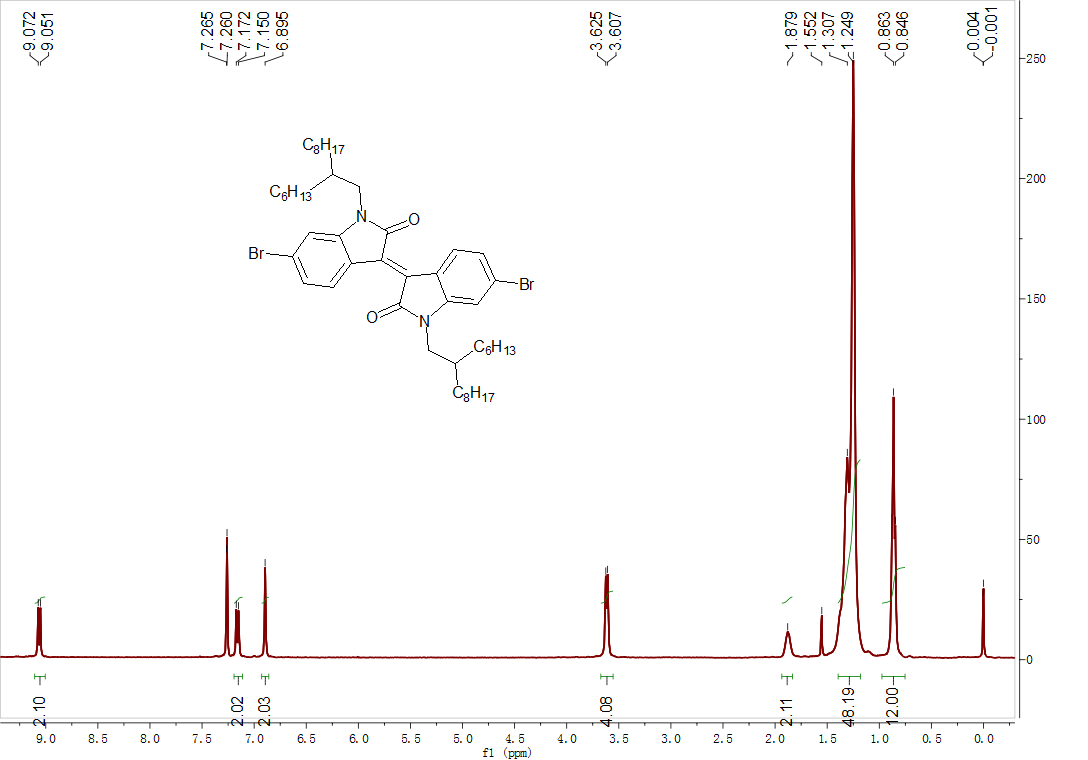


**Figure S2.**^1^H-NMR spectrum of M2 in CDCl_3_ solution.


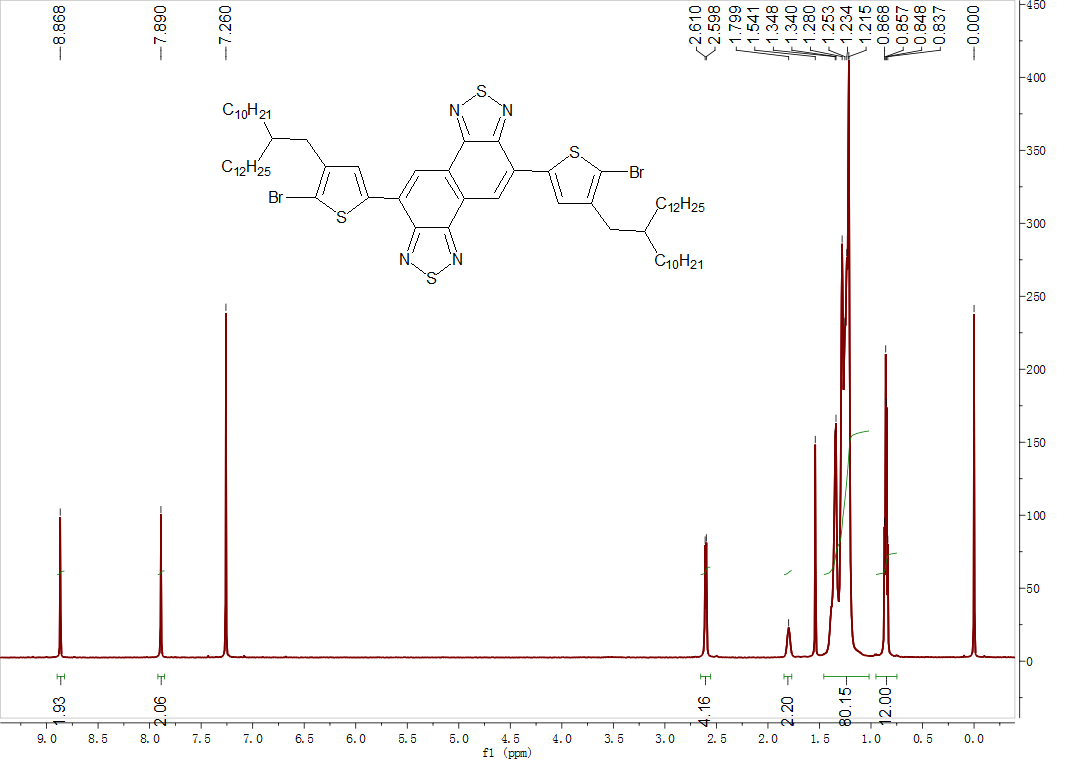


**Figure S3.**^1^H-NMR spectrum of M3 in CDCl_3_ solution.

2. X-Ray Diffraction Patterns ofBlend Films forthePolymers.


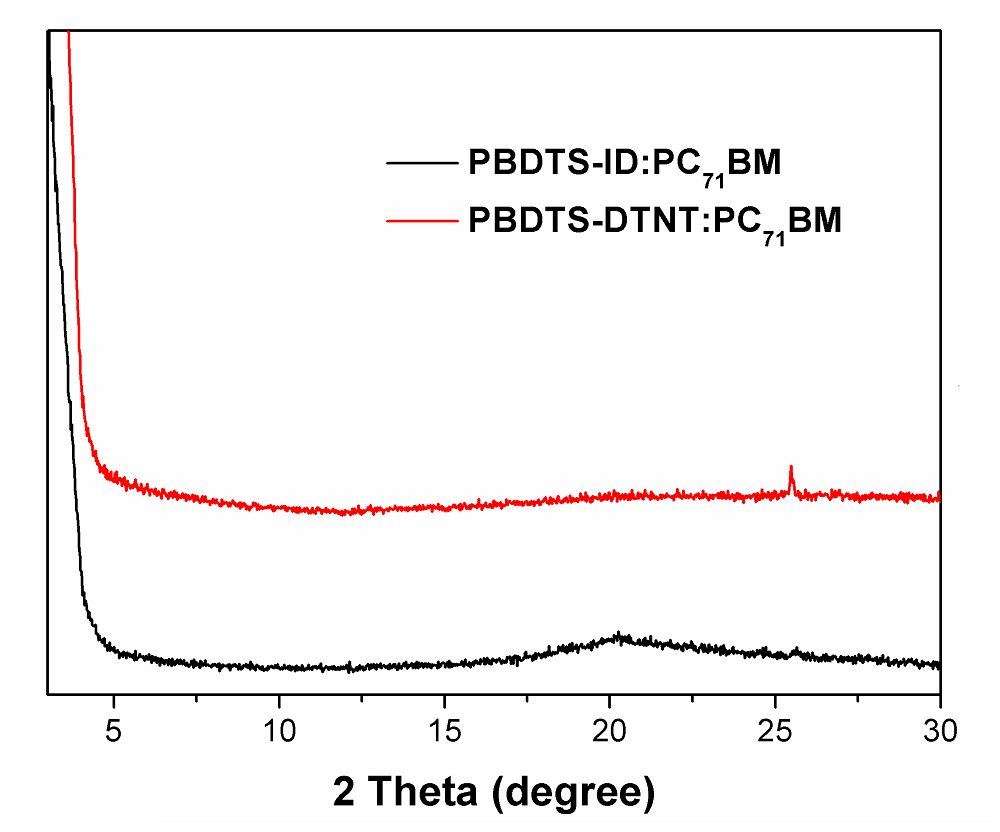


**FigureS4.** X-ray diffraction patterns of blend films for the polymers.
